# Decolonial process tracing: Indigenous rights and pipeline resistance movements

**DOI:** 10.1177/26349825231154872

**Published:** 2023-02-15

**Authors:** Sakihitowin Awasis

**Affiliations:** Western University, Canada

**Keywords:** Process tracing, Indigenous rights, energy governance, decolonial theory, anishinaabe studies

## Abstract

This article explores how decolonial methodologies and Anishinaabe *gkendaasowin* (ways of knowing) can augment detailed narrative process tracing methodologies used to examine social and political processes. While detailed narrative is most frequently used as a tool of causal inference, focusing on the unfolding of a singular time, I see potential for it to be enriched by Indigenous legal traditions that emphasize epistemic diversity and multiple temporalities. Analyzing how Indigenous rights are leveraged in decision-making processes for the Line 9 and Line 3 pipelines, I show how a decolonial approach to process tracing (DPT) that centers Anishinaabe *gkendaasowin* can change both the actors and power relations involved. Recognizing that energy decision-making processes take place alongside, outside, and within colonial state institutions, and are embedded in the land as constellations of reciprocal kinship responsibilities, DPT opens space to examine two kinds of Indigenous rights: those acquired through struggle with state institutions, and those inherent to Indigenous communities’ attachment to place. DPT addresses the shortcomings of a focus on linearity by privileging inherent rights that are often excluded from detailed narrative process tracing. To take inherent rights seriously, one must also take more-than-linearity and more-than-humans seriously—and DPT is uniquely positioned to do this. The key features I propose for decolonial process tracing are grounded constellations, multiversality, and multitemporalities. Decolonizing methodologies and Anishinaabeg studies provide direction for more expansive, decolonial process tracing techniques which can in turn help understand the relationship between temporalities, law, and energy governance.

## Introduction

If you have ever watched a lake freeze for the winter, you may have noticed how nonlinear processes of ice formation are. Ice may form at the edges, melt, reform, crack, open in mosaic, freeze, weaken, refreeze, and eventually solidify into a foundation that can be traveled and fished upon. This process is different for each lake, each year, yet relies on the same complex network of interconnected processes: lake size, flow, precipitation, wind velocity, solar radiation, and snow cover, among others (Bengtsson, 2012a, 2012b). The body of water, sun, clouds, wind, and snow are all interrelated in inherent ways that contribute to the outcome of lake ice. Lakes have also acquired processes from human activity that impact ice formation, including industry, wastewater treatment, and climate change. Inherent and acquired processes often interact in ways that comprise an asymmetry of power. In the Great Lakes region, for instance, climate models predict reduced ice cover in a 30-year period due to human greenhouse gas emissions (Byun and Hamlet, 2018).

Social processes, including decolonial processes that seek to undo the ongoing impacts of settler colonialism, are also nonlinear—yet most research into decision-making relies on linear analysis. Hall (2003) notes a “growing misalignment of methodology and ontology in the social sciences” and “how we think the world actually works may not fit with the methods we use to study it.” Process-tracers aim to be sensitive to complex interactions, the specific institutional and historical contexts in which processes unfold, background factors that are often omitted, as well as the spatiotemporal scope of mechanisms and the theories underpinning them (Falleti and Lynch, 2009; Morgan, 2016; Trampusch and Palier, 2016). This calls for attention to nonlinear processes and systems theory, such as feedback loops, choice theory, social network analysis, missed opportunities, and critical junctures (Beach, 2016). However, this attention often does not go far enough in affirming the existence of multiple, distinct spatiotemporalities that provide for Indigenous self-determination, communities’ capabilities to choose their/our own decision-making, and governance structures.

Indigenous pipeline resistance movements, a widespread and growing form of decolonial action, involve several forms of nonlinearity, including constellations of place-based kinship networks across multiple spatiotemporalities (many past, present, and future (non)human worlds). Constellations of Indigenous people and nonhuman kin on the frontlines of antipipeline struggles interact with multiple legal orders, including two kinds of Indigenous rights that reflect asymmetrical power relations: those inherent to Indigenous communities’ attachment to place, and those acquired through struggles with colonial state institutions. Indigenous pipeline resistance movements are re-actualizing governance systems built around inherent rights and land-based responsibilities. Across *Mikinaak Minis* (Turtle Island or North America) there has been ongoing Indigenous-led opposition to pipeline projects since the Idle No More movement began in the winter 2012. Indigenous law and land-based knowledge systems feature strongly in these movements. Notably, the Unist’ot’en House of the Gilseyhu Clan, and the Gidimt’en and Laksamshu Clans of the Wet’suwet’en nation are challenging seven proposed oil and gas pipeline projects across Wet’suwet’en Yintah (territory) by establishing healing camps and checkpoints, as well as pursuing legal action through both their hereditary clan governance structures and colonial courts (Unist’ot’en Camp, 2017). Mass Lakota-led resistance refused the Keystone XL and the Dakota Access Pipelines (Estes, 2019). Along the proposed Trans Mountain pipeline route, the Scwepemec Tiny House Warriors (2020) have built 10 tiny homes to assert Secwepemc law and jurisdiction, and an alliance of Anna’s hummingbirds and Tsleil-Waututh community members halted construction for 4 months during the Summer 2021 nesting season (Judd, 2021). These examples point toward the shortcomings of process tracing and the need for more expansive, decolonial approaches. Constellations of humans and nonhumans are rebuilding mechanisms for shared decision-making in ways that amplify place-based relationships (Daigle and Ramírez, 2019; Simpson, 2017). Indigenous bodies on the frontlines of antipipeline struggles cogenerate interdependent theory, praxis, and processes of governance in interrelationship with the land (Simpson, 2011, 2017, 2021).

Yet, research documenting such Indigenous-led movements usually frames energy governance and energy justice in relation to colonial governments’ laws, institutions, and processes, and privileges linear interpretations of causality and time. Process tracing, my focus here, includes a variety of techniques used to examine how people and organizations make decisions. It is a laborious undertaking that demands the careful review of large volumes of documents in the form of proposals, institutional mandates, intervenor evidence, impact assessments, panel reports, council resolutions, constitutions, policies, interview transcripts, and other sources such as media reports. Process tracers also pay attention to the actions of and interactions between people and organizations, as well as contextual factors, that shape social and political outcomes (Bengtsson and Ruonavaara, 2017). Process tracing can be used to carefully examine the context and contents of laws, assess areas of divergence between two legal systems, and go beyond “what” or “why” questions to better understand the “how” of law (Farrand, 2020). However, dominant process tracing methodologies are often marked by assumptions about the linearity of time, universality of knowledge, and the problematization of equifinality (the existence of multiple causal pathways that can lead to an outcome of interest).

In this paper, I propose ways to expand and enrich detailed narrative process tracing—to make it more decolonial—by engaging with insights from Anishinaabe gkendaasowin (ways of knowing). My aim is to provide process-focused theoretical interventions that could contribute to more just and sustainable energy decisions by decentering anthropogenic authority and reinforcing Indigenous legal systems as central to self-determination in energy decision-making. I focus on detailed narrative forms of process tracing due to their compatibility with Anishinaabe gkendaasowin. Anishinaabe gkendaasowin are embodied, dynamic, and complex knowledge systems specific to Anishinaabeg peoples and generated from “doing” in a direct relationship with the land (Pine, 2016). Anishinaabe gkendaasowin is also embedded in *Anishinaabemowin*, the Anishinaabe language, and provides meaning that cannot be translated into English (Corbiere, 2013).

These methodological reflections grew out of my work on Anishinaabeg pipeline resistance (Awâsis, 2020a, 2020b) which I contend is not merely a protest movement, but a conflict between divergent legal systems and ways of knowing. I draw examples from Anishinaabeg resistance to the expansion of Line 9 (Sarnia, Ontario to Montréal, Québec) and Line 3 (Hardisty, Alberta to Superior, Wisconsin). During the 2012-2017 Line 9 pipeline dispute, Deshkan Ziibiing Anishinaabeg (Chippewas of the Thames First Nation) challenged the Line 9 decision in the Supreme Court of Canada, citing the Crown’s failure to consult the nation. There has also been active and ongoing Anishinaabe-led resistance to Line 3 since the 2014 proposal. The Minnesota Chippewa Tribal Government (2017) conducted their own public hearings and cumulative impact assessment on Line 3 in response to inadequate consultation through the Public Utilities Commission (LaDuke, 2015). Consistent with decolonial and resurgence theory, I am less interested in why pipelines result from linear, colonial decision-making processes. Instead, I focus on how constellations of Anishinaabeg peoples decide to oppose pipeline projects within Anishinaabe legal systems.

Natural law resides in nature and is derived from the legislative power of the land (Ferreira da Cunha, 2013). Decolonial theory and Natural law are both deeply informed by what the land, as a system of reciprocal relationships and responsibilities, can teach us about living in the world in nonhierarchical and nondominating ways (Borrows, 2018; Coulthard, 2014). Colonialism is not experienced as a historical event that negatively impacts the present, but as hierarchical gender, racial, and class structures that are maintained by a series of complex and overlapping processes (including treaty making, law making, reconciliation, consultation, impact assessments, and court systems) (Mignolo and Walsh, 2018; Simpson, 2017). Processes of both settler colonialism and decolonization are complex and multifaceted, comprised of overlapping processes that require more expansive analytical approaches to understand.

To bridge the disciplinary boundaries between decolonizing and process tracing methodologies, I ask: (1) In what ways are dominant forms of process tracing limited when engaging with Indigenous rights? And (2) how can process tracing be more decolonial? I begin by briefly exploring how process tracing has thus far relied on theorizations of a linear temporality, universality, and equifinality. I bring process tracing methodologies into dialogue with key themes from decolonizing methodologies and Anishinaabeg studies, including constellations of nonhuman actors and multiple spatiotemporalities. Using examples from Lines 9 and 3, I explore how Anishinaabeg pipeline opponents decide which rights to leverage because this distinction between inherent and acquired rights makes explicit the interactions between linear and constellated temporal formations. Finally, I examine the significance of decolonial process tracing techniques and propose future directions for decolonial process tracing scholarship. I conclude that decolonial process tracing has the potential to open further opportunities for Indigenous legal systems by centering inherent rights and nonhumans in decision-making.

## Dominant forms of process tracing and their limitations

Process tracing aims to understand mechanisms of change by characterizing causal and temporal processes that led or lead to an outcome of interest (Trampusch and Palier, 2016). Process tracing techniques were first developed in cognitive psychology in the late 1970s to study individual decision-making. Political economy and international relations scholars have since applied process tracing techniques to identify intervening steps that influence organizational decision-making and the effects of institutional arrangements on processes and practices (Bennett and Checkel, 2014; George, 1979; George and Bennett, 2005; George and McKeown, 1985). Today, process tracing techniques are applied to a variety of issues across disciplines (Farrand, 2020). While analyzing mechanisms as theoretical processes is not unique to process tracing and many social scientists use variants of process tracing without referring to it as such, the terminology has become more common since the mid-2000s (Kittel and Kuehn, 2013; Morgan, 2016).

While all process tracing involves movement back and forth between empirical evidence and theory, its many forms can be grouped into two categories: those that are more inductive (aimed at theory building) versus deductive (aimed at theory testing) (Trampusch and Palier, 2016). Inductive approaches include detailed narrative process tracing, the focus of this article. Detailed narrative forms explore causal ideas embedded in a narrative along a timeline of events (Collier, 2011; George and Bennett, 2005). Detailed narrative process tracing is distinct from pure narrative because it focuses on specific aspects of a phenomenon, its structure is based on a theoretical framework, and it aims to explain a causal path leading to a specific outcome (Vennesson, 2008). While process tracing originated to analyze decision-making processes, it has become predominantly used to compensate for weaknesses in correlational analysis and improve causal identification and inference (Beach, 2016; Morgan, 2016).

However, correlations provide little insight into causal mechanisms, and correlational analysis is based on deeply held assumptions about linear temporality and unit homogeneity that are so central to explanation that many political scientists and sociologists simply take them for granted. For example, one could wrongly assume that all members of an Indigenous community have the same priorities and desire the same governance structure. While process tracers identify how factors or phenomena relate to each other, a persistent problem in process tracing literature is that many social, political, and ecological phenomena of concern such as power, rights, inequality, institutions, violence, and participation are embedded in dynamics that do not always make causal identification possible. Identifying and explaining processes can be especially difficult in social systems and require significant prior methodological reflections (Hay, 2016).

Trampusch and Palier (2016) offer a list of good process tracing practices that include clarifying research assumptions, selecting good theory, drawing on a variety of data sources, conducting thought experiments, and practicing transparency. They also suggest that process tracers who have a nondeterministic conceptualization of mechanisms and do not apply statistical analysis face a substantial challenge in further developing methodological concepts and standards. This article contributes to this methodological task using a decolonial lens. Process tracing is versatile enough to examine change among different legal systems, making it possible to describe energy decision-making in Anishinaabeg legal contexts. However, even when best practices are carefully followed, several limitations remain that diminish its applicability to Anishinaabeg contexts. I now explore three specific limitations of dominant process tracing methodologies: linearity, universality, and the problem of equifinality.

### Linearity

As a tool of causal inference, dominant forms of process tracing focus on the unfolding of events over linear time and describe events at isolated points along this singular axis (Collier, 2011). This colonial temporality relies on homogeneous movement through empty time in “a successive series of presents, each becoming past in turn” (Rifkin, 2017: 17). A 7-day work week, 24-hour clock time, and the Gregorian calendar, as well as Canadian and American institutions are all based on assumptions about a linear temporality (Huebener, 2015). This temporality frames decision-making as a successive line of development and is central to capitalist modes of production and operation (Castree, 2009). Concepts such as progress, productivity, acceleration, instantaneity, and simultaneity underlying the linear temporal structure can be considered a form of timespace compression. Timespace compression is a set of processes through which the spread of technologies such as the Internet and smartphones effectively shorten spatiotemporal distances, or in some cases, eliminate their relevance altogether (Harvey, 1989). To increase profit and the speed at which commodities are produced and circulated, settler capitalism has a drive for timespace compression; this includes infrastructure such as pipelines that increase the speed at which fossil fuels reach markets (McCreary, 2020). The temporal qualities of settler capitalism require and normalize the dispossession of Indigenous peoples from ancestral lands, governance structures, and nonlinear temporalities to secure control of land and labor for exploitation.

The linearity of the colonial temporal logic limits Indigenous governance and reinforces colonial power relations in several ways (Huebener, 2015). Settler authorities are provided with the power to define the starting points, ending points, and duration of social processes. The present and shorter durations are prioritized over other timeframes. Finally, constraints are imposed on temporal modes based on race, age, gender, class, culture, sexuality, and nationality. Dominant forms of process tracing are both limited and limiting when they do not account for lived temporalities that exceed the linear formation. Along the linear temporality, Indigenous peoples get plotted in ways that deny temporal multiplicity and mobility that are inherent in Indigenous social and cultural life as well as political resurgence. In dominant pipeline reviews, for example, colonial decision-making authorities invalidate Indigenous self-determination and flatten Indigenous governance by excluding Indigenous place-based and nonhuman temporalities that serve as the background for Indigenous political and economic systems (McCreary, 2020; McCreary and Milligan, 2013). Against the linearity of the colonial, institutional background, Indigenous governance is treated as a difference within colonial nations, and Indigenous lands are required to remain open to capitalist expansion.

In colonial decision-making, Indigenous governance is flattened (dissociated from social, political, ecological, and economic claims) and frozen (fixed to the past) (Borrows, 2012; Coulthard, 2014). For instance, Indigenous rights are frozen when colonial authorities demand that Indigenous governance, land title, and land-based activities demonstrate linear continuity with practices that were happening at the time of contact or effective control to be formally recognized, through Section 35 of the 1982 Constitution Act in Canada and the 1831-1832 Marshall decisions in the United States (Borrows, 2002, 2012). The fixation on precontact practices ties Indigenous peoples to the past and severely restricts Indigenous legal systems (Huebener, 2015; Rifkin, 2017). Colonial courts maintain the power to violate Indigenous rights on grounds that they do not demonstrate linear continuity (Ogden, 2009). In colonial decision-making, “tradition” is only regarded as “authentic” when it emerges from the time of colonial contact/control and, with a linear progression of time, the distance between present-day Indigenous peoples and the past source of authenticity is steadily increasing (Richotte, 2013). By embracing a linear conception of time, process tracing reinforces the authority of settler institutions and precludes Indigenous ways of knowing and modes of governance. Decolonial process tracing can support Indigenous self-determination by accounting for a multiplicity of temporalities.

### Universality

Dominant forms of process tracing assume epistemic universality (the singularity of the knowledge system) and universal synchrony (participation in a mutual now). The notion of a singular and all-encompassing time in which all events unfold relies on the naturalization of a shared present as if it were a neutral and self-evident medium. Conceiving of time as a universal line of development entails that it is possible to assign every event, person, place, and thing to a span on the continuous flow of a global timeline.

The assumed universal nature of time has several consequences. Insisting on epistemic universality is a way of reinforcing the dominant knowledge system and perpetuating ontological racism (Mignolo and Walsh, 2018: 205), stemming from colonial assumptions about Indigenous peoples holding a subontological position in academia, governance, and everyday life (Ciccariello-Maher, 2017; Sundberg, 2014). Indigenous systems of order have been dismissed, and Indigenous ways of knowing are viewed as not human, civilized, literate, or adequate (Smith, 2013). As a result, while dominant forms of process tracing emphasize methodological plurality, they do not give adequate attention to epistemological plurality. Despite their differences, positivism, constructivism, and critical theory—widely used in process tracing—all treat knowledge as singular in nature (Wilson, 2008). Process-tracers often make the multiculturalist assumption that we exist in a world with many different cultural understandings of a single nature, while Indigenous knowledge systems entail multinaturalist ontologies (Coombes et al., 2012) and the pluriversal (Mignolo and Walsh, 2018), recognizing the existence of many worlds.

The universality of the colonial temporal logic functions to normalize the temporality of the settler state and impose it on Indigenous communities. Temporal assimilation is integral to settler colonialism. In the 19th century, Indian legislation, such as the Indian Act (1876) in Canada and Indian policy (1810s–1890s) in the US, homogenized Indigenous nations into colonial forms of governance, fragmented Indigenous confederacies into reserve (Canada) and reservation (US) boundaries (Irwin, 1997; Simpson, 2017), criminalized gatherings and ceremonies, delineated Indian status on non-Native terms, facilitated large-scale dispossession, and enforced attendance at residential schools (Canada) and boarding schools (US). Colonialism violently disconnects Indigenous peoples from our histories, homelands, languages, social relations, and knowledge systems (Smith, 2013). The assumed universality of the normative temporal structure undermines and assimilates Indigenous ways of knowing and living, reinforcing colonial power. Settler time equates Indigeneity with backward relations to “real” time, framing Indigenous nations as in need of colonial institutions to make objective decisions on their behalf (Huebener, 2015; Rifkin, 2017). This colonial power relation is reflected in process tracing’s overreliance on human-made law and acquired Indigenous rights. Decolonial process tracing can challenge colonial power when grounded in inherent Indigenous rights and Natural law.

### Equifinality

Process tracing literature points to “the problem of equifinality”—sometimes also called “multiple causality”—when the possibility that there are multiple pathways that may lead to the same outcome is perceived as a problem (Farrand, 2020). Dominant forms of process tracing seek to account for equifinality by considering alternative explanations for a mechanism and determining which explanation is more likely to have resulted in the effect (Bennett and Checkel, 2014; George and Bennett, 2005). Alternatively, deciding to embrace equifinality rather than “resolve” it can shift how we understand causality and power. For example, Guzzini’s (2017) interpretivist conception of causality sees relationality as characterized by equifinality: the same effect (if a, then b) can result from several different pathways from a to b. This is typical of social processes; a configuration of multiple paths often contributes to an outcome. Mechanisms can be part of, but are not reducible to, a wider process that can help answer “how” questions (Guzzini, 2017). The challenge of a relational configuration is thinking of causation in terms that seem to be contradictory, like openness and indeterminacy.

A relational approach to understanding power undermines dominant process tracing’s problematization of equifinality. Power is a capability that exists in and through relation, not as an event nor possession of any agent prior to the relationship in which it is exercised. We cannot explain “power” without knowing the context of the relation and the people sharing it (Guzzini, 2017). There is no necessary causal line from Indigenous rights policy and discourse to a single understanding and action, yet process-tracers can demonstrate which possible capabilities were excluded. Without specific capabilities, certain pathways or processes, like self-determination, could not happen. Guzzini (2017) proposes using “social mechanisms” to frame a version of causation that addresses the open process, the mobilization of capacities/abilities, intersubjective mechanisms, and the reality of equifinality. In this sense, the causal reconstruction of mechanisms can be characterized by multicausality and nonlinear processes. The mechanism itself depends on its interaction with other mechanisms and the process in which it unfolds.

Similarly, the Minnesota Chippewa Tribe’s (2017) Cumulative Impact Assessment describes how, in traditional ecological knowledge systems:

there is a more holistic understanding of cause and effect. There is an understanding that cause A and effect B cannot be isolated from cause B and effect B in a system. This is known as “mutualistic logic” and “reciprocal causality.” In practical terms, this is the difference between examining the increase of GHG from a pipeline project (by direct emission, replacement increases, etc) and examining the impact increased investment in fossil fuel infrastructure will have on future generations.

Decolonial process tracing embraces relationality and reciprocal causality. With this conceptualization in mind, the next section details Anishinaabe gkendaasowin (ways of knowing) as a framework for understanding social processes. I use the critique just presented as motivation to develop a view of process tracing that does not rely on linearity and universality, nor problematize equifinality. The role of causality and the settler state move to the periphery. The questions informing the next section are: How can process tracing be more decolonial? How do Anishinaabe understandings of the relationality of place and power reroute process tracing? To begin, I will introduce the idea of mechanisms as communities’ capabilities. Then, I will illustrate how such a form of social mechanisms can be used in decolonial analysis, focusing on grounded constellations, multiversality, and multitemporalities.

## Gkendaasowin

Indigenous self-determination in decision-making demands an expansive understanding of spacetime that does not treat settler institutions as the baseline for Indigenous governance and does not treat multiplicity as weakness. I see possibilities to enrich process tracing by engaging with multiple (including nonhuman) spatiotemporalities and ontologies. Anishinaabe gkendaasowin provides a guiding framework for this decolonial project as a congenial approach outside the process tracing tradition. It is not possible to summarize the complexity of Anishinaabe knowledge systems here, nor do I have the authority to do so. Instead, I highlight the breadth of decolonizing methodologies that range from detailed family narratives (Daigle, 2018) to statistical associations demonstrating community support for kinship-based governance (Jewell, 2018). Anishinaabe gkendaasowin can provide explanations in ways that are both consistent with and contradict positivist and constructivist assumptions. My engagement with gkendaasowin and decoloniality is not meant to be comprehensive but focused on some of the processes and capabilities for Indigenous self-determination that are embedded in detailed narratives.

Biskaabiiyang is an Anishinaabe understanding of decolonization as individual and collective processes of embodying freedom and returning to ourselves and the land (Geniusz, 2009; Simpson and Manitowabi, 2013). In this sense, decolonial processes involve communities’ capabilities to engage in deep and reciprocal land-based relationships and responsibilities (Corntassel, 2008; Kimmerer, 2015) that comprise the basis of Indigenous political systems, economies, and nations (Coulthard, 2014). Centering Anishinaabe gkendaasowin, Chi Inaakonigewin (Natural law) extends to dodemiwan (clan governance) (Jewell, 2018). Dodemiwan is a series of overlapping consensus-based decision-making structures comprised of extended kinship relations with animal nations, and less commonly plant and other nonhuman nations, that provide both social identity and function. Dodemiwan is grounded in land-based power, existing across distinct spatiotemporalities, in a decentralized system generated and maintained by Anishinaabe people in direct relationship with the land. The structural and material bases of pre/decolonial Indigenous life was/is process centered (Coulthard, 2014; Simpson, 2017). For Anishinaabe scholar Basil Johnston (1991), Anishinaabe is a verb and a noun; it is something we are and something we do at the same time. Anishinaabe ways of living and processes of governance are “both the instrument and the song” of self-determination—the goal as well as how we get there (Simpson, 2017: 19). Michi Saagiig Nishnaabeg scholar, Leanne Betasamosake Simpson (2017) considers diverse Indigenous ways of living on our homelands “the primary mechanism” for decoloniality (p. 21).

The compatibility of capabilities theory and critiques of colonial power relations make a communities’ capabilities approach appropriate for framing mechanisms for Indigenous self-determination in decision-making. Sen (1999) and Nussbaum (2006)’s theory of justice concentrates on the capacities necessary for individuals to lead the kinds of lives that they freely choose for themselves and have reason to value. The focus of justice is not on the distribution of resources, but how goods are transformed into the capacity for human flourishing, and how injustices disrupt or limit what people can do or be. Schlosberg and Carruthers (2010) elucidate a pluralistic, community-centered capabilities approach to development based in Indigenous communities’ diverse notions of environmental justice. Unlike liberal political thought, Indigenous environmental justice movements do not limit themselves to understanding injustice as individuals, and often situate their struggles in collective experiences of injustice that impact communities’ ability to function (Schlosberg and Carruthers, 2010). Community-based definitions of capabilities that are central to Indigenous environmental justice struggles can be integrated into a concern for the basic functioning of ecosystems, intergenerational knowledge, and the cultural, political, and spiritual life of communities (Schlosberg and Carruthers, 2010). Indigenous demands for equity, recognition, and participation are incorporated into a larger concern for the basic functioning of socioecological communities. This is akin to promoting mino bimaadiziwin, or “a good way of life” in Anishinaabemowin, which can be understood as the mutual flourishing of natural and cultural communities (Doerfler et al., 2013. In discussion with Simpson (2011), Anishinaabe Elder Robin Greene roots environmental sustainability in the process of mino bimaadiziwin . . . “so that life can promote more life” (p. 141).

Indigenous communities’ capabilities can be understood as real opportunities to choose how decisions are made. Sen (1999) makes an important distinction between functioning and real opportunities by drawing on an example of two people, one fasting and one starving. An affluent person who is fasting may function the same as a person living in poverty, in terms of not eating, but the affluent person has a different capability set of alternatives from which to choose, in this case, to access food to eat. We can attach importance to having real opportunities, even when they are not acted upon, in the same way, we can distinguish fasting from starving. Sen acknowledges that choosing itself is a valuable functioning, and the process through which outcomes are generated has its own significance. Indigenous communities’ capabilities to choose their governance structures and the underlying temporalities make self-determination possible. Whether a community then decides to translate these general capabilities “to choose” into more specific capabilities (e.g. clan governance, elected band council, or a hybrid), is up to the grassroots people. Capabilities theory does not dictate function: the evaluative focus is not on realized functionings (what someone actually does or what governance structure communities actually choose), but the capability set of alternatives (real opportunities for self-determination, or for communities to choose their own governance structures). Process tracing approaches encourage analysis of process(es) in which such capabilities do and do not exist (Guzzini, 2017). For example, when there is a violation of Indigenous law through the imposition of a pipeline project, are Indigenous communities capable of upholding legal decisions made through clan governance? In the case of Deshkan Ziibiing Anishinaabeg, Jewell (2018) provides statistical evidence that the majority of her community supports clan governance. What is of central importance to decolonial process tracing is whether Indigenous nations have community capabilities, real opportunities to deliberate and build consensus in traditional, hereditary, and grassroots councils (Manuel and Derrickson, 2017). Sen’s concept of capabilities provides for a deeper, more context-sensitive analysis of justice issues associated with energy decision-making (Bickerstaff et al., 2013).

What decolonization means and entails differs depending on where you are and can include layered understandings of decoloniality in the same place (Tuck and Yang, 2012). Decolonial process tracing demands that mechanisms are derived from multiple ontologies, open systems, and nonlinear processes. Many Indigenous people regard the starting and ending points of a sequence as less important than the ongoing process of relationship building. Reciprocal recognition can be considered an inherent Anishinaabeg place-based practice that promotes relationship building (Simpson, 2017). I do not use the term reciprocal recognition in a Hegelian sense. Instead, reciprocal refers to the act of recognizing and being recognized by a living, animate landscape. Reflecting recognition back to the land as a reciprocal political practice and affirmation of dignity can be understood as part of Natural law that promotes mino bimaadiziwin (Kimmerer, 2015; Simpson, 2011). Simpson (2017: 184–185) explains how Basil Johnson uses the term maamaayawendamoowin to describe the process of reciprocal recognition: the first maa meaning “it’s in my heart,” maamaaya meaning “fully understanding yourself or another being,” and wendamoowin meaning “your thought process as you move through life.” Simpson’s teacher, Doug Williams, distinguishes maamaaya from baamaaya, which refers to “searching for recognition,” presumptively from colonial authorities, which I interpret as more akin to the Hegelian notion of recognition.

Maamaayawendamoowin is a process of seeing another’s essence and amplifying reciprocity in place-based relationships. Anishinaabeg reciprocal recognition is embodied in our everyday lives when we ground ourselves in the web of land-based relationships that give us meaning. Indigenous self-recognition is about presence in our bodies and on the land; recognition that our bodies are created and sustained through shared relationships of deep reciprocity with and responsibilities to human and non-human collectives, communities, and nations (Simpson, 2017). Both terms for recognition, maamaayawendamoowin and baamaayawendamoowin, include the word, wendamoowin, which promotes remaining rooted in Anishinaabe thought processes as we move through life, and while we engage in both reciprocal and colonial forms of recognition. This is important because when Indigenous peoples engage with non-Indigenous institutions, it is often a struggle to retain connections with our relatives; it is common to start to feel removed from our grounded relationships and disconnected from intuitive ways of doing things in the community (Manuel and Derrickson, 2017; Wilson, 2008). It is important how recognition happens and whether communities have real opportunities to participate in the governance structures they choose and value. Self-determination is an ongoing process that relies on Indigenous communities’ direct relationship with the land, involves committed engagement between humans and nonhumans, and includes the communities’ capability for the everyday embodiment of inherent responsibilities (Corntassel, 2008; Kimmerer, 2015). To do this, Anishinaabe gkendaasowin transcends the colonial linear temporality, dissolves the problem of equifinality, and emphasizes both methodological and epistemological diversity. Below I articulate related opportunities for decolonial approaches to process tracing: grounded constellations, multiversality, and multitemporalities.

### Constellations

The basis of a constellation is the relationship, between humans and nonhuman beings across spacetime (Simpson, 2017). Decoloniality is not a lineal point of arrival; in a constellation, there are many routes through spacetime and many ways to embody structures and relationships centered on relational accountability (Daigle and Ramírez, 2019). Recall how the formation of lake ice depends on the interrelationship between the unique body of water, sun, clouds, wind, snow, and human activity. We can trace how lake ice coverage is declining over time because of a shift in the constellation of relationships that privileges human industries over the inherent ways lake ice governs themselves. Constellations of small collectives’ organizing rooted in gkendaasowin provide real opportunities to rebuild governance using Natural law and clan decision-making processes. Individuals with common goals come together to make decisions and act, then disperse or reform, and continue to develop relationships with other collectives (Simpson, 2017). Constellated formations are the embodied knowledge of Indigenous people in direct engagement with the land, including urban spaces, and other dispossessed and oppressed peoples. This is also how Indigenous pipeline resistance movements are built and operate: as constellations of human and nonhuman collectives creating mechanisms for communication, accountability, and shared decision-making.

McCreary (2020) argues that rather than focusing on the concentration and accumulation of wealth, the continual renewal of kinship relations in constellation with human and nonhuman kin through gift-giving promotes place-time extension. While commodity exchanges within capitalist systems disrupt Indigenous social relations, gift-giving generates community and social connection. This is also situated within Indigenous understandings of intergenerational responsibility, stretching reciprocal obligations into the past and future in ways that are open-ended and integral to one’s identity. Kinship here includes shared responsibilities within and between clan families, and across species, it is not the same as biological descent or immediate family relationships (Whyte, 2021). These nonlinear kinship relations are responsive and adjust when there are disruptions.

In Anishinaabeg resistance to Line 3, the White Earth Nation unanimously enacted the Rights of Manoomin (“the good seed” or wild rice) in December 2018, recognizing wild rice within the White Earth territory has the inherent right to exist and flourish (White Earth Band of Minnesota Chippewa Tribe, 2018). These rights are brought into action in the name of Manoomin themselves, as the real party of interest, and central actor in legal interventions to protect their rights from violations along the proposed pipeline corridor. The rights of Manoomin are far-reaching and include the right to freshwater habitat and the right to a healthy climate. This highlights the plurality of self-determining nonhuman persons in Anishinaabe pipeline resistance, as well as how national-level governance is embodied in constellations of local place-based relationships. Although decolonial process tracing engages with actors in institutional decision-making, relationships that exist beyond institutions are prioritized. Anishinaabe gkendaasowin instigates wide intellectual engagement that centers place-based ways of living (Simpson, 2017).

An integral component of decolonial process tracing is the consideration of the multiplicity of land-based relationships and how these are tied to specific sites and embodiments of self-determination. Constellations are in formation and emerging all around us every day, across struggles for self-determination, peoples rooted in different places are renewing relationships in their/our own communities. These unique histories and geographies that generate formations of constellations in co-resistance with each other and the land guide us toward decolonial futures (Daigle and Ramírez, 2019).

### Multiversality

While universality is the logic of colonialism, multiversality is the basis of many different movements for decolonization (e.g. Indigenous Revolutionary Clandestine Committee-General Command of the Zapatista Army of National Liberation, 1996). “Where do you begin telling someone their world is not the only one?” (Maracle, 1993: 72). Multiversality (or pluriversality) describes the existence of many spatiotemporally distinct yet interconnected worlds (Cajete, 2000). Mignolo and Wash (2018) describe how pluriversal decoloniality “opens rather than closes the geographies and sphere of decolonial thinking and doing” (p. 3). It interweaves local histories, subjectivities, knowledge systems, narratives, and struggles against colonialism and for self-determination.

In the Minnesota Chippewa Tribe’s (2017) Cumulative Impact Assessment on the proposed Enbridge Line 3 Expansion and Abandonment Plan, the Anishinaabeg world is described as: “eight planes of existence, with an understanding of the deep relationship between the time of the ancestors and the time of the descendants.” Constellations seek to open up and advance radically distinct perspectives and positionalities that displace western scientific rationality as the only possible framework for analysis, encouraging a more relational way of living and inviting us to think with nonhuman peoples, struggles, and knowledge systems. A fundamental aim of decoloniality is the revitalization of epistemic diversity (Mignolo and Walsh, 2018). Decolonization is a process of refusing or delinking from the colonial matrix of power to regenerate pluriversality. Recalling Simpson’s (2017) queer normativity, knowledge is not a single set of ideas—it is the interaction between many different meanings, including discord, that make sense within Anishinaabe gkendaasowin. “You see, there are always worlds on top of worlds, worlds, underneath worlds, worlds intertwined with worlds. It’s a sort of Nishnaabeg String Theory” (Simpson, 2021: 30). Approaching knowledge as place-based is a decolonial necessity to relocate colonial universals in multiple forms of local emergence and restore them to their local scopes (Mignolo and Walsh, 2018: 205).

### Multitemporalities

Indigenous narratives of time can exceed the dominant temporality in a variety of ways, including: prophecies, the presence of past ancestors and future descendants; land-based kinship political systems; queerness; memories; ancestral land-based knowledge including attunement to nonhuman and climatic temporalities; intergenerational knowledge and stories as a basis for engaging with people, places, and nonhumans; situating events within a much longer timeframe (generations, centuries, or millenia), and responsibilities to past and future generations (Rifkin, 2017). “[the pluriversal] opens up coexisting temporalities kept hostage by the Western idea of time and the belief that there is one single temporality” (Mignolo and Walsh, 2018: 3). Temporality is not a way to measure or calibrate processes; rather, the processes themselves involve multiple spatiotemporalities (Rifkin, 2017). Attending to temporal multiplicity means resisting broad static typologies, openness to internal forms of difference, and movement between categories in dynamic ways (Rifkin, 2017). Temporal multiplicity in decolonial process tracing means shifting from a shared now toward a deeper consideration of what constitutes distinct temporal formations and how these different formations engage and interact with each other.

Without being plotted on a singular timeline, Anishinaabe temporal multiplicity comes from direct engagement with the land as kin and is inherent in a natural flow of time—the creation of the multiverse, the rounds of the seasons, the lunar cycle, and the earth’s rotation. There are no abstract or universal concepts of “time” in Anishinaabemowin. Related terminology carries greater specificity: *aabiding* (at one time), *azhigwa* (at this time), or *gomaapii* (for some time) (Richotte, 2013). Simpson (2017) describes an Anishinaabe understanding of time coded in the etymology of the word biidaaban (dawn): the prefix “bii” refers to the coming future, the verb “daa” means being present in a specific place, and the verb “ban” is used when someone no longer exists (p. 193). The past, present, and future coexist in Anishinaabe temporal understandings; not only does the past influence the present and future, but desired futures shape how we engage with the past and present. Similarly, aanikoobijigan refers to both ancestor and descendant, suggesting the existence of past and future relatives, as well as interaction with the actual and/or potential actions of our human and nonhuman ancestors and descendants (Whyte, 2021). Multitemporalities animated by Indigenous lands and not dissected in past, present, and future open immense possibilities for Indigenous self-determination.

Decolonial process tracing concentrates on the more-than-institutional temporal contexts within which communities build decision-making power. In the face of ongoing settler colonialism, expressions of Indigenous self-determination lie in the capability to participate in decision-making processes that do not take the colonial temporality and state as the implicit context. Constellated networks of relationship—stories, ceremonies, and the land itself—are procedures for decision-making (Simpson, 2017). It is critical not to assume here that Indigenous stories take place exclusively in ancient times. Traditional stories have the ability to apply to Anishinaabe peoples in multiple temporal settings, presume the present existence of past and future (non)humans, and maintain a deep sense of continuity that connects the past, present, and future (Nadasdy, 2008). Anishinaabe spatiotemporalities are not only cyclical in nature (same species, different individuals) but also circular (same individuals in the present, past, and future). This distinction is important for understanding how Anishinaabe people engage with temporal multiplicity; human and nonhuman nations exist in the present through our past and future inter-social relations. The past, present, and future societies of humans and nonhumans all exist in a continuous interrelationship.

There is a both an opportunity and responsibility for decolonial process tracing to address power imbalances between settler and Indigenous societies, as well as human and nonhuman societies in our characterizations of energy decision-making processes by tracing constellations outside colonial institutions, in addition to Indigenous interactions with linear processes. Decolonial theory directs process tracers toward reciprocal responsibilities and relational accountability that are sustained through all stages of research, including topic selection, data collection, analysis, review, and dissemination. The next section applies insights from decolonizing methodologies and Anishinaabe gkendaasowin to detail methodological considerations that are integral to a decolonial approach to process tracing.

## Biskaabiiyang and process tracing

Decolonial process tracing emphasizes personal relationship building and visiting with human and nonhuman communities at every stage of the research process. Topic selection that is guided by relational accountability and reciprocity directs us to responsibilities to the land and local community members. The proposed topic should be identified based on community need and support, strength-based, solution-focused, and sensitive to community history and how meaning is embedded in local landscapes (Louis, 2007). The research topic can offer a means of restoring humans’ relationships with more-than-human worlds. My relational accountability and responsibility to the land and community in topic selection extends from reclaiming Indigenous names and placenames, and honoring community protocols, to reframing my research approach based on feedback from community members that I should prioritize inherent rights.

To examine detailed narratives of how Anishinaabeg people understand Indigenous rights in pipeline decision-making, I offered tobacco to Anishinaabeg people who have been involved in resistance to the Line 9 and/or Line 3 pipelines and invited them to visit with me while we shared a meal or refreshments when possible. Sometimes this amounted to an interview one-on-one or in a small group that was open-ended and dialog based to provide for the mutual sharing of information through focused discussion (Wilson, 2008). Participants for both case studies live along the Line 9 or Line 3 pipeline routes but descend from communities across Anishinaabe Aki (territory), including from Ojibwe, Potawatomi, Odawa, Oji-Cree, Algonquin, Mississauga, Nipissing, Saulteaux, and Métis nations. Although decolonial process tracing prioritizes inherent rights and thus inherent rights holders who are from communities that are located along the pipeline route, Anishinaabeg pipeline resistance movements are comprised of the anikoobijigan of many (non)human nations. My decision to invite Anishinaabeg peoples from communities across the homelands to participate was to help challenge settler colonial divisions of Anishinaabe territories and bodies into Canada/US, reserve/urban, and status/non-status dichotomies, as well as to strengthen reserve-rural-urban and cross-border relationships. Decolonial process tracing that reflects these aims and considerations while centering the inherent rights and concerns of local communities can meaningfully contribute to nation-building efforts.

Dominant forms of process tracing implicitly encourage a focus on questions of what is or what ought to be Indigenous rights, comprising a normative approach to the study of Indigenous governance that predominantly focuses on colonial forms of recognition and colonial decision-making structures. Instead, decolonial approaches refocus attention on how rights are embodied in diverse ways in praxis and value communities’ capabilities to choose inherent governance structures. Dominant forms of process tracing are often carried out in theory-informed, yet empirically open-ended ways and the techniques are considered most fruitful if stories are generated in terms and questions suggested by theory, but not limited to it (Bengtsson and Ruonavaara, 2017). Indigenous rights scholarship is often over-determined by theory focused on acquired rights. Centering colonial institutions and forms of recognition undermines Indigenous legal systems and limits community-based decision-making processes by positioning the state as the locus of legitimate decision-making power.

Indigenous rights discourses are embedded in Anishinaabe pipeline resistance movements and pipeline opponents’ stories in which political focal points can be identified and analyzed. Focal points can be understood as key conjunctures where the restricting role of the colonial institution is made explicit. Focal points are good indicators of where linear processes can be expanded upon in a constellated form. For example, Wiindmaagewin (Consultation Protocol) Deshkan Ziibiing Anishinaabeg passed in November 2016 begins by stating that the purpose of the law is to protect their watersheds, relationships, and rights. Theories of energy justice and temporal justice, as well as ongoing personal relationships with participants, guided my analysis of in-depth interviews, evidence presented at hearings and in court challenges, and the constitutions, consultation protocols, and bylaws of Anishinaabeg nations. These complex configurations allowed for comparison and further examination using a holistic, systems approach. Shawn Wilson (2008), in conversation with Peter Hanohano, discusses

An analogy that Peter once used is that the data and analysis are like a circular fishing net. You could try to examine each of the knots in the net to see what holds it together, but it’s the strings between the knots that have to work in conjunction in order for the net to function. So any analysis must examine all of the relationships or strings between particular events or knots of data as a whole before it will make any sense. (p. 120)

The relationships that connect distinct elements of the multiverse are important (Cajete, 2000). It’s not enough to theoretically decenter humans or map out relationships, it is something you do through embodiment and living, building more relations, being active on the land and in community. Theorizing alone is not gkendaasowin; knowledge must be applied in praxis.

Anishinaabe people contextualize and embody energy governance practices through our respective communities’ laws and political principles, and pipeline resistance movements are helping to regenerate these political and legal orders. The transformative potential of Anishinaabe pipeline resistance movements serves to influence both general understandings of law and specific approaches to energy justice. Resurgence “isn’t just a recognition of the complexity and multidimensionality that we might not fully understand at work. It is also a strategic, thoughtful process in the present as an agent of change” (Simpson, 2017: 20). Indigenous pipeline resistance movements emphasize structural transformation as pathways to deep decarbonization and Indigenous self-determination that are not apparent through dominant process tracing approaches.

For decolonial process tracing, the processes underlying how freedom is attained should be of central importance. In colonial contexts, injustice can work through freedom rather than exclusively against it, and *how* we attain capabilities determines whether real opportunities are present or not. Recalling Sen’s (1999) distinction between fasting and starving, or capacities and opportunities, Indigenous peoples and settlers may have the same functioning in terms of freedom to participate in an elected government; however, in colonial contexts, Indigenous peoples’ participation in electoral politics can also function as an “unfreedom” if there is not a real opportunity to choose a traditional form of governance. This is why Simpson (2017) calls for mobilizing around Indigenous systemic alternatives, because “how we live, how we organize, how we engage in the world–the process–not only frames the outcome, it is the transformation” (p. 19). Re-actualizing Indigenous systemic alternatives radically changes our ways of knowing and living, the actors involved, our modes of production, and how we embody governance.

Decolonial process tracing is concerned with whether Indigenous nations can determine the processes with which to pursue freedom. Building on Sen’s (1999) understanding that among different functionings, some are more effective at promoting freedom than others, I conclude this section by exploring a particular unfreedom, or contradictory functioning of freedom in colonial contexts. A concern brought up by multiple participants during the Line 9 interviews was the question of whether Indigenous nations should accept funding from the colonial government to participate in consultation. This directly relates to the Supreme Court ruling on Line 9, which noted as part of its decision to dismiss community objections to the consultation process that “Chippewas were granted funding to participate in the process” (Supreme Court of Canada, 2017). Community members expressed the worry that accepting government funds intended to enhance Indigenous communities’ capabilities to participate in energy decision-making signifies to colonial authorities that consultation has occurred, despite consultation processes that remain largely impartial or nonexistent. For Deshkan Ziibiing Anishinaabeg, accepting state funding to ostensibly enhance freedom may have impacted their community’s capabilities to challenge the adequacy of consultation in court. The funding came with limits on what communities can do (Manuel and Derrickson, 2017). When situated in the ethical frameworks of Natural law, energy decision-making processes can improve environmental outcomes and promote Indigenous freedoms. However, when situated in market or colonial frameworks, there is a risk that outcomes will maintain injustice by undermining Indigenous social and environmental goals at a future date. Community capabilities that expand freedom at one time can limit it at another. This highlights that decolonial process tracing should be sensitive to unfreedoms, the contradictory role freedom can have over time in colonial contexts, and possible tradeoffs between present and future capabilities.

Decolonial process tracing insists that communities have the actual opportunity to shape their lives and energy decision-making processes, not just be passive recipients of ready-made state-centered development programs. The land and local Indigenous communities themselves should be the source of decision-making power for grassroots structures that provide real opportunities for community deliberation, mobilization, and embodiment of governance practices (Borrows, 2002; Manuel and Derrickson, 2017).

## Conclusion

At the time of writing, it is Manidoo-Giizoons (Little Spirit Moon or December) in the Great Lakes. Recently, while walking along the Kagawong river on M’Chigeeng Anishinaabeg territory near where I am living, I was visiting with the salmon migrating from Odaawawi Gichigami (Lake Huron) to spawn. With awe-inspiring strength, they hurl their bodies upstream against strong downward currents, flipping in the air, over obstacles, slapping against the surface, and chasing each other. I was startled when the path led me to one of the largest Salmon I have ever seen, struggling in the shallows beside me. It was a jarring sight, her jawbone was exposed, she had large sores down her sides, her tail was frayed, and her gills seemed to be slowly falling apart. The Salmon had put all her energy into the difficult trip upriver and reproducing, and as a result, was disintegrating in front of me.

I conclude with this story because several ideas within it are important to understanding decolonial process tracing. First, like swimming upstream to reproduce and decompose, it is largely difficult and unglamorous, what Simpson (2017) calls the “hard work of being present”; building lodges, cooking food, caring for elders and children, making mistakes, and organizing with our people, even when we disagree, are all practices that form the basis of Anishinaabeg political and economic systems. Second, decolonial process tracing is also concerned with temporal concepts such as communities’ intergenerational health, healing, and equity. Intergenerational health and healing relate to the biological, cultural, and social reproduction, and communities’ capabilities to heal from colonial violence, respectively. Intergenerational equity pertains to fairness between past, present, and future generations. Like other Salmon before her, the Salmon I saw is literally giving her body to provide nutrients for the next generation, as well as aquatic insects that can also be eaten by young Salmon. In both her life and death, the adult Salmon helps promote the flourishing of her species and the land and waters as a constellation of interrelationships. Third, there is a direct connection between the Salmon’s body and the surrounding environment. Decolonial process tracing engages with Indigenous governance and law that are embodied, embedded in the land, and more expansive than colonial law. Fourth, Indigeneity cannot easily be equated with local (Castree, 2004). Most of the salmon in the Great Lakes today are Pacific Salmon that were relocated from their ancestral territories and were introduced into the Great Lakes from 1966-1970, and they now outnumber the local Atlantic salmon (Parsons, 1973). Similarly, although Indigenous pipeline resistance movements center local communities’ rights and concerns, many Indigenous people today live in urban centers and/or do not live on their ancestral territories. In Indigenous-led pipeline resistance movements, decolonial process tracing is a useful tool to better understand ourselves and the lands on which we live from within local Indigenous governance systems. In brief, DPT seeks to make Indigenous ways of knowing more central to process tracing by providing context for a more expansive understanding of Indigenous law, governance, and rights. This demands an openness to constellations of decision-making processes, many human and nonhuman worlds, and multiple temporalities.

While this paper has laid a conceptual foundation for decolonial process tracing, it does not provide a template to apply concepts or a set of best practices. I invite others to share how they “use the fishing net,” build relationships, and “do” decolonial process tracing to help build rich, flexible, and collective understandings of DPT in praxis that prioritize local communities of inherent rights holders, challenge settler colonialism, and recenter land as the source of decision-making power.
